# What Makes Mice Fat? How the Brain Controls Energy Balance

**DOI:** 10.1371/journal.pbio.0030438

**Published:** 2005-11-29

In most animals, food intake and energy expenditure vary greatly from day to day. Yet, in healthy young animals, cumulative energy intake over several days matches energy use very closely. This balancing act or “energy homeostasis” is controlled by complex neuronal circuitry and numerous signaling molecules. When these control mechanisms go wrong, the result is weight loss or obesity. Middle-aged spread, for example, is probably caused by a progressive impairment of energy homeostasis.

Two types of neurons in the hypothalamus—a region deep in the brain that controls many aspects of physiology—help to regulate fat buildup, or adiposity. First, there are proopiomelanocortin (Pomc) neurons, so called because they make proopiomelanocortin. This is a precursor for melanocortins, peptides that bind to melanocortin receptors elsewhere in the brain to limit food intake and increase energy expenditure. Then there are agouti-related protein (Agrp) neurons. These make agouti-related protein, named for its similarity to a protein mutated in an obese mouse with a characteristic yellow coat. By blocking melanocortin receptors, Agrp increases food uptake—the scientific term for this is hyperphagia—and decreases energy use. Both types of neurons detect circulating indicators of body adiposity such as leptin, and then act to keep energy stores constant. [Fig pbio-0030438-g001]

**Figure pbio-0030438-g001:**
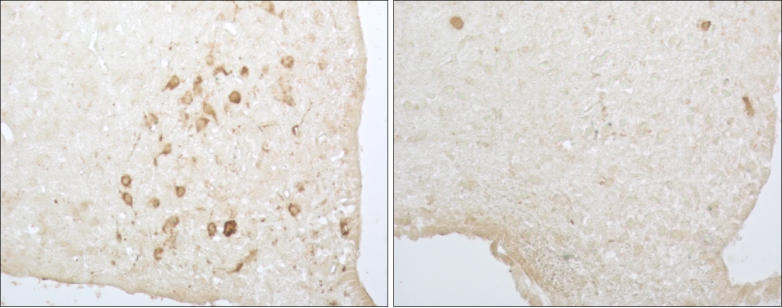
Normal mouse hypothalamic tissue (left) and mutant tissue (right), with Pomc neurons deleted

Support for this model for energy homeostasis comes from rodent studies in which the hypothalamus was damaged or stimulated, or in which leptin and other peptides were injected directly into the brain. Genetic experiments in mice provide further support but also some conflicting evidence. While deletion of the Pomc gene or overexpression of Agrp increase appetite and obesity as predicted by the model, unexpectedly, deletion of the Agrp gene does not disturb energy balance. One explanation for this is that Pomc and Agrp neurons might play a role in energy homeostasis even when they don't express their defining peptides; it is known, for instance, that Pomc and Agrp neurons express additional neuropeptides with effects similar to Pomc and Agrp, and that Agrp neurons control the activity of Pomc neurons.

To investigate more fully the roles that Pomc and Agrp neurons play in energy homeostasis, Allison Wanting Xu et al. have constructed mouse strains in which the Pomc or Agrp neurons are lost progressively after birth. They took advantage of a technique that selectively deleted the gene for the mitochondrial transcription factor A *(Tfam)* in Pomc- or Agrp-expressing neuronal cells. *Tfam* is required for transcription of the mitochondrial genome, which encodes proteins required for cellular respiration and thus cell survival. By six months old, the researchers report, the engineered mice had lost many of their Pomc or Agrp neurons but no other neurons.

Like aging humans, mice in which Pomc neurons had died became progressively fatter because of an increased food intake and reduced energy expenditure. Mice that had lost Agrp neurons weighed slightly less than control animals, and mice engineered so that both types of neurons died weighed more than control mice but less than those lacking just Pomc neurons. These results indicate that the regulation of adiposity by Pomc and Agrp neurons is not simply a matter of releasing these two neuropeptides.

Xu et al. made an additional, unexpected observation. After food deprivation, mice normally increase their food intake acutely until their fat stores return to prefasting levels—a process called compensatory hyperphagia. Paradoxically, mice without Pomc neurons showed reduced compensatory hyperphagia despite overeating under normal conditions. Since aging humans also fail to increase their food intake after fasting, these mouse strains that gradually lose specific hypothalamic neurons provide a potentially informative model of human age-related obesity. In addition, by studying such mice, scientists may gain important insights into the full complexity of how hypothalamic neurons regulate energy balance that could help to reverse the current human obesity epidemic. —*Jane Bradbury*

